# Serogroup W Meningitis Outbreak at the Subdistrict Level, Burkina Faso, 2012

**DOI:** 10.3201/eid2111.150304

**Published:** 2015-11

**Authors:** Laurence Cibrelus, Isaïe Medah, Daouda Koussoubé, Denis Yélbeogo, Katya Fernandez, Clément Lingani, Mamoudou Djingarey, Stéphane Hugonnet

**Affiliations:** World Health Organization, Geneva, Switzerland (L. Cibrelus, K. Fernandez, S. Hugonnet);; Ministère de la Santé, Ouagadougou, Burkina Faso (I. Medah, D. Koussoubé, D. Yélbeogo);; World Health Organization Intercountry Support Team for West Africa, Ouagadougou (C. Lingani, M. Djingarey)

**Keywords:** meningococcal meningitis, serogroup W meningococcal meningitis, outbreak, epidemic, Burkina Faso, vaccine, meningococcal conjugate vaccine, MenAfriVac, Sub-Saharan Africa, policy, Meningitis Belt, immunization, bacteria, Neisseria meningitides

## Abstract

In 2012, *Neisseria meningitidis* serogroup W caused a widespread meningitis epidemic in Burkina Faso. We describe the dynamic of the epidemic at the subdistrict level. Disease detection at this scale allows for a timelier response, which is critical in the new epidemiologic landscape created in Africa by the *N. meningitidis* A conjugate vaccine.

Since 2010 in sub-Saharan Africa, a meningococcal A conjugate vaccine (MenAfriVac; http://www.meningvax.org/) has been widely introduced to at-risk areas in the meningitis belt, resulting in a change in the epidemiology of meningococcal meningitis in the region (*1*,*2*). Fewer meningitis cases are now diagnosed, and large outbreaks of *Neisseria meningitidis* serogroup A are diminishing. Proportionally, more epidemics are caused by other meningococcal serogroups (e.g., serogroup W) that have less salient epidemic patterns, limiting detection through district-level surveillance and delaying intervention measures (*3*). World Health Organization guidelines for outbreak response in sub-Saharan Africa were revised in 2014 partly to address this issue (*4*). To ensure timelier intervention, the guidelines recommend that epidemic risk be assessed for populations of 30,000–100,000.

In 2010, MenAfriVac was introduced in Burkina Faso (*5*), and meningitis incidence was low in 2011; however, during February–April 2012, several epidemic foci occurred at the district level (*6*). This epidemic was the first in Burkina Faso since introduction of MenAfriVac and the second serogroup W epidemic in the country, occurring 10 years after the initial outbreak (*7*,*8*).

Meningitis outbreak dynamics are seldom studied on a small scale (e.g., at the subdistrict level). However, key information for improving detection of and response to epidemics can be learned from such analyses. To add to the current knowledge, we studied meningitis outbreak dynamics in areas of Kombissiri district, Burkina Faso, that were most severely affected by the 2012 epidemic. 

## The Study

The study was a collaborative effort of the Disease Control Department of the Burkina Faso Ministry of Health and the World Health Organization. District-level aggregated surveillance data (weeks 1–17, 2012) and official population data were used. At the subdistrict level, population and surveillance data (weeks 1–16, 2012) were collected from the Kombissiri district surveillance unit. Standards for meningitis surveillance were used (*9*). The weekly alert and epidemic status for Kombissiri and its subdistricts were determined by using the 2012 meningitis incidence thresholds and the 2014 revised guidelines, which have a lower alert threshold (*4*,*9*).

**Figure 1:**
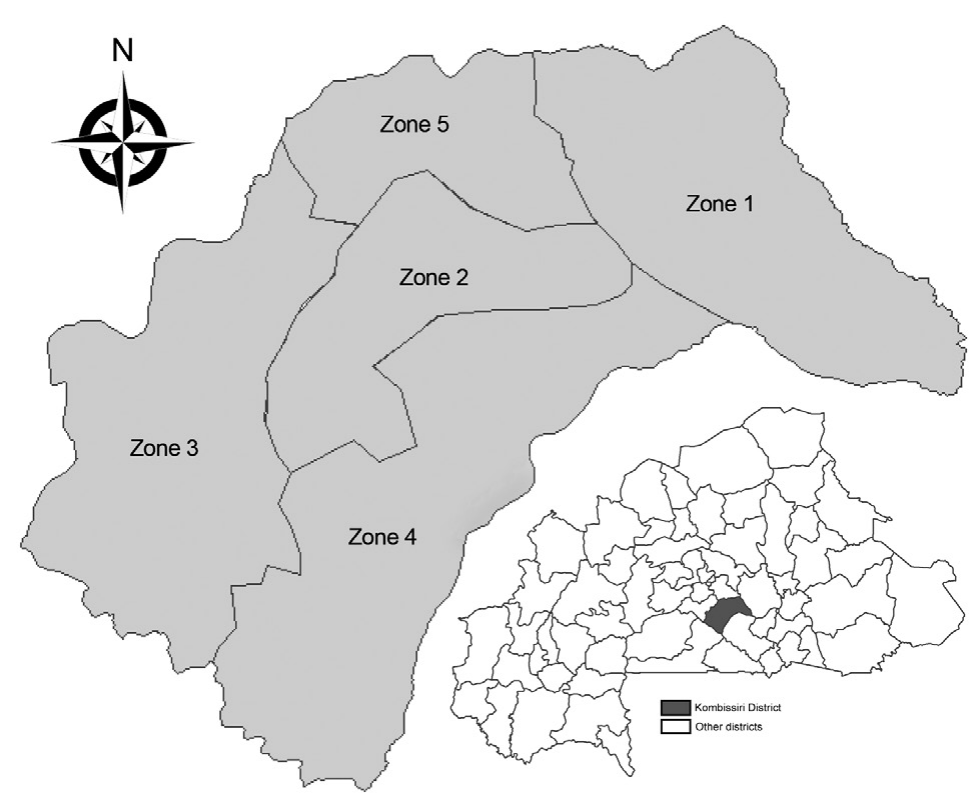
Zones within Kombissiri district, Burkina Faso.

In 2005, subdistrict zones were created in Burkina Faso to improve disease surveillance accuracy and timeliness; districts were subdivided into zones of ≈30,000 persons (Table). Within these zones, population numbers are similar, unlike numbers in health facility (HF) catchment areas, which in Kombissiri range from 440 to 25,000 persons. In Burkina Faso, zone data are infrequently analyzed, except in Kombissiri (Figure 1). 

**Table:** Details of the 2012 outbreak of *Neisseria meningitidis* serogroup W at the district and subdistrict (Kombissiri district) levels, Burkina Faso

Outbreak level	Population	Duration, wk (starting wk)			Time, wk, to peak**††	Attack rate, no. cases/100,000 population	
		Alert plus epidemic*†	Preepidemic‡§	Epidemic¶#		Weekly maximum‡‡	Cumulative§§¶¶
District, epidemiologic wks 1–17							
Banfora	312,923	4 (11)	1	1 (12)	0	10.5	62.0
Bittou	116,080	8 (8)	5	3 (13)	1	20.7	92.2
Dafra	285,184	8 (8)	4	3 (12)	2	16.8	96.4
Dande	225,917	6 (9)	3	3 (12)	1	12.0	70.8
Gourcy##	196,686	5 (12)	3	1 (15)	0	10.2	62.5
Kombissiri	173,885	9 (7)	4	3 (11)	1	14.4	105.2
Orodara	346,319	7 (9)	2	4 (11)	2	16.2	91.2
Pama	98,308	7 (9)	4	1 (13)	0	15.3	89.5
Po	185,632	6 (10)	3	1 (13)	0	10.2	51.2
Sapone	96,020	6 (9)	5	1 (14)	0	11.5	67.7
Seguenega##	189,363	7 (11)	3	1 (14)	0	12.1	60.7
Sindou	147,477	7 (9)	1	4 (10)	2	15.6	89.5
Solenzo	314,593	7 (8)	0	5 (8)	2	16.2	101.7
Kombissiri District, epidemiologic wks 1–16							
Zone 1	39,163	10 (6)	2	7 (8)	3	38.3	153.2
Zone 2	32,037	15 (1)	2	13 (3)	10	46.8	293.4
Zone 3	34,591	1 (12)	Not applicable	Not applicable	Not applicable	5.8	14.5
Zone 4	30,541	2 (12)	0	1 (12)	0	13.1	39.3
Zone 5	37,553	9 (6)	5	1 (11)	0	10.7	39.9

*Defined as time between weekly attack rate crossed at least the alert threshold (5 cases/week/100,000 population) and descended below the alert threshold, (i.e., from alert to alert). †Mean 6.7; median 7; SD 1.32. ‡Defined as time between weekly attack rate crossed the alert threshold and reached the epidemic threshold (10 cases/week/100,000 population) (i.e., from alert to epidemic). Not applicable if only the alert threshold was crossed. §Mean 2.9; median 3; SD 1.55. ¶Defined as time between weekly attack rate crossed the epidemic threshold and descended below the epidemic threshold again (i.e., from epidemic to epidemic). Not applicable if only the alert threshold was crossed. #Mean 2.4; median 3; SD 1.45. **Defined as time between weekly attack rate crossed the epidemic threshold and reached maximum incidence (i.e., from epidemic to peak). A zero value indicates peak was reached when the epidemic threshold was crossed. Not applicable if only the alert threshold was crossed. ††Mean 0.8; median 1; SD 0.9. ‡‡Mean 14.0; median 14.4; SD 3.9. §§Over the study period. ¶¶Mean 80.0; median 89.5; SD 18.0. ##A reactive immunization campaign with ACWY polysaccharide vaccine was conducted during week 18, 2012.

On a national level, 13 (21%) of the 63 districts in Burkina Faso reached meningitis epidemic status in 2012 (Table; Technical Appendix). *N. meningitidis* serogroup W caused 82.6% (384/465) of confirmed cases in these districts, in which no reactive vaccination against this serogroup had been recently conducted. Kombissiri had the highest cumulative attack rate (CAR) and was affected longer than other districts. Of 136 suspected meningitis cases in Kombissiri, 44 (32%) were confirmed: 36 (82%) of those were caused by *N. meningitidis* serogroup W, 1 (2%) was caused by *N. meningitidis* serogroup X, and 7 (16%) were caused by *Streptococcus pneumoniae*. District- and subdistrict-level distributions of these pathogens were comparable.

**Figure 2:**
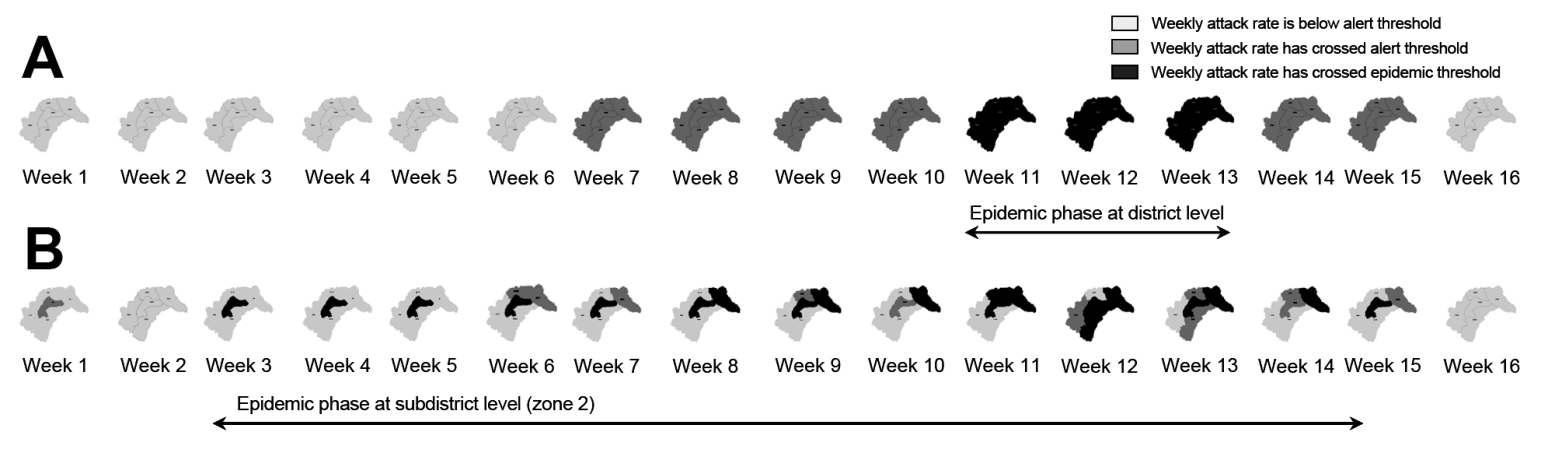
Weekly meningitis alert status and epidemic status at the district (A) and subdistrict (zone) (B) level in Kombissiri district, Burkina Faso, during epidemiologic weeks 1–16, 2012. The alert threshold was 5 cases per week per 100,000 population. The epidemic threshold was 10 cases per week per 100,000 population.

The outbreak in Kombissiri reached alert and epidemic thresholds during weeks 7 and 11, respectively; a total of 9 weeks were spent in these phases (Table). At the subdistrict level, alert and epidemic thresholds were reached at weeks 1 and 3, respectively, 6 and 8 weeks, respectively, earlier than at the district level. Alert and epidemic thresholds were first crossed in zone 2, and contiguous zones were gradually affected from week 6 onward (Figure 2). Zones 1 and 2 (outbreak epicenters) were the only zones in the epidemic phase for >2 continuous weeks. In these zones, the average alert and epidemic phases were longer than those at the district level (12.5 vs. 9.0 weeks), the preepidemic phase was shorter (2.0 vs. 4.0 weeks), the time to peak was longer (6.5 vs. 1.0 weeks), and the epidemic phase started earlier (week 3 [zone 2] and week 8 [zone 1] vs. week 11) and was longer (10 vs. 3 weeks) (Table). The average CAR and peak incidence in zones 1 and 2 were higher than those for Kombissiri (CAR 223.3 vs. 105.2 cases/100,000 population; peak incidence 42.6 vs. 14.4 cases/week/100,000 population) and other districts (Table). The levels were also higher than those in 2012 serogroup W epidemics with district-level documentation in The Gambia (CAR 111 cases/100,000 population) and Benin (CAR 123.7 cases/100,000 population; peak incidence 16.7 cases/week/100,000 population) (*10*,*11*). If the 2014 recommendations for epidemic detection had been used in Kombissiri, the district-level preepidemic phase would have been 4 weeks longer, reaching the alert threshold in week 3 rather than 7 (13 weeks total in alert and epidemic phases). At the zone level, in the outbreak epicenter, the alert phase would have begun 1 week earlier in zone 1; no change would have been seen for zone 2, which was in the alert phase since week 1.

## Conclusions

During the 2012 serogroup W meningitis epidemic in Burkina Faso, localized subdistrict epidemics occurred before those identified at the district level. Subdistrict epidemics were also of longer duration and greater intensity. At the subdistrict level, the epidemic spread from the 2 epicenter zones to other contiguous zones; several zones were affected before the district reached epidemic status.

If a subdistrict-level epidemic risk assessment had triggered district-level interventions in Kombissiri, meningitis surveillance and microbiologic testing could have been intensified (alert phase) and epidemic control measures could have been implemented (epidemic phase) up to 6 and 8 weeks earlier, respectively, by using epidemic response guidelines in use at the time and up to 2 weeks earlier by using 2014 guidelines. At the district level, the short time between crossing the epidemic threshold and attaining the epidemic peak (average <1 week) leaves a limited window for effective intervention because reactive immunization has a moderate effect on natural epidemic evolution after an epidemic has peaked (*12*). In this epidemic, the time between crossing the epidemic threshold and reaching peak was much longer when characterized at the zone level (3 and 10 weeks, respectively, in the 2 outbreak epicenters). Characterizing the epidemic risk at the subdistrict level with the 2014 alert threshold for a population of ≈30,000 yielded critical time gains, particularly during the epidemic preparedness phase. Early interventions with longer implementation windows improve response efficiency and might have halted this epidemic before it spread throughout Kombissiri. Timelier, targeted interventions could potentially be mounted at district or even subdistrict levels if the epidemic risk is localized. Too little evidence is available now to consider modifying the epidemic threshold; lowering the threshold might trigger unnecessary resource-intensive interventions, using limited vaccine supplies. However, modifications should be reconsidered once dynamics of non–serogroup A meningitis epidemics are better understood at the subdistrict level (*4*).

The advantages of reducing the spatial scale of epidemic risk assessment were recognized when *N. meningitidis* A was driving the epidemiology of meningitis in Africa and phenomena detected at HF level developed into large epidemics (*13*,*14*). However, outbreaks caused by non-A meningococcal meningitis serogroups have less resonant patterns, and analysis of the epidemic risk at a level within the subdistrict (e.g., HF level) may lack sensitivity (*4*). Nevertheless, it is essential that good quality data be readily available at that level for finer analysis when needed. The infrequent use of subdistrict-level data is partly due to their limited routine availability. Collection of subdistrict-level data was difficult when enhanced surveillance was the predominant approach for surveillance in the meningitis belt, but the transition toward case-based strategies (according to which meningitis cases are individually described at the HF level) should help fill this gap (*9*,*15*).

Technical Appendix Epidemic and alert districts during epidemiologic weeks 1–17, 2012, Burkina Faso.
