# Supplementary material for: Serogroup W Meningitis Outbreak at the Subdistrict Level, Burkina Faso, 2012
Source: Emerg Infect Dis. 2015 Nov;21(11):2063–6. doi: 10.3201/eid2111.150304 (PMC4622241; doi:10.3201/eid2111.150304)
Supplement: Technical Appendix — Epidemic and alert districts during epidemiologic weeks 1–17, 2012, Burkina Faso. [file 15-0304-Techapp-s1.pdf]

Article DOI: DOI: http://dx.doi.org/10.3201/eid2111.150304

Serogroup W Meningitis Outbreak at the Subdistrict Level, Burkina Faso, 2012

Technical Appendix

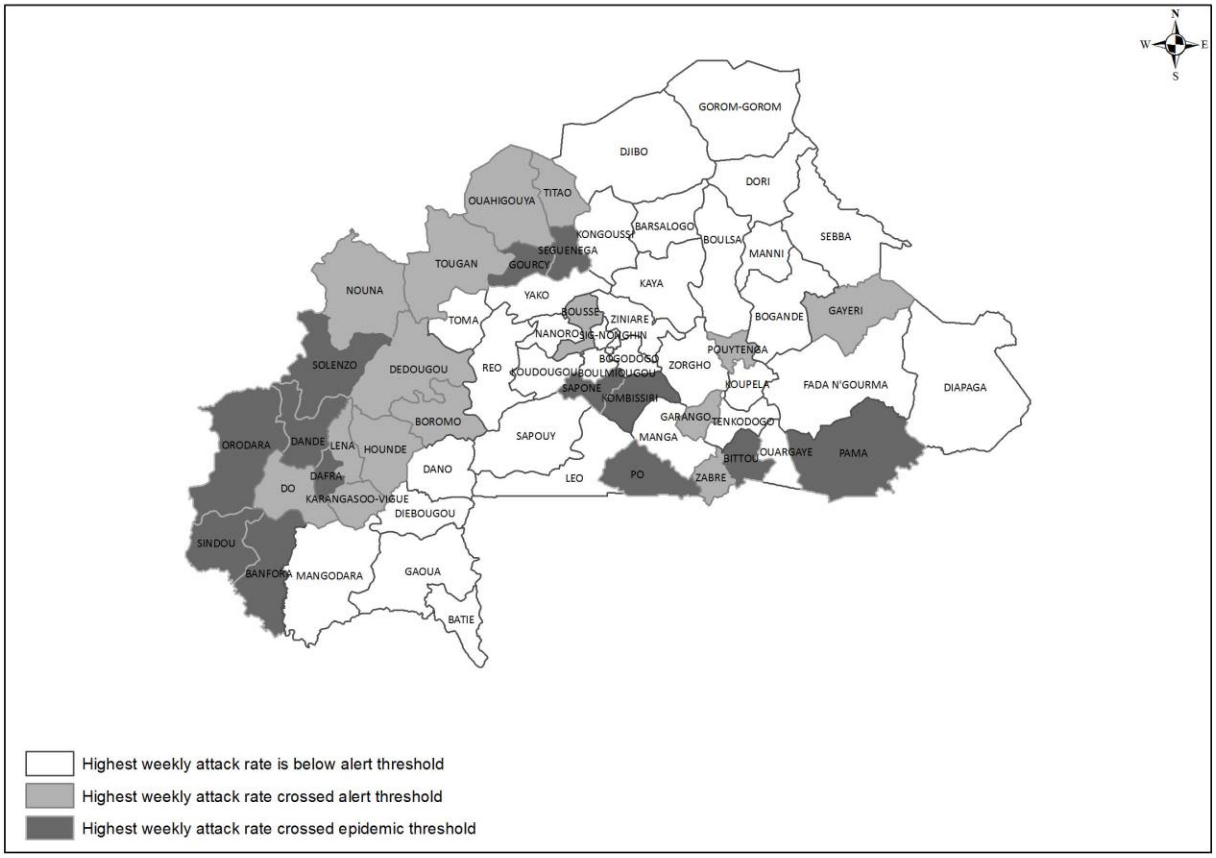

Technical Appendix Figure. Epidemic and alert districts during epidemiologic weeks 1–17, 2012, Burkina Faso. The alert threshold was 5 cases per week per 100,000 population. The epidemic threshold was 10 cases per week per 100,000 population.
